# Knowledge and Practices towards Prevention and Early Detection of Chronic Kidney Disease and Associated Factors among Hypertensive Patients in Gondar Town, North West Ethiopia

**DOI:** 10.1155/2020/2860143

**Published:** 2020-08-06

**Authors:** Daniel Asmelash, Elias Chane, Getahun Desalegn, Sewmalet Assefa, Getie lake Aynalem, Alebachew Fasil

**Affiliations:** ^1^Department of Clinical Chemistry, School of Biomedical and Laboratory Sciences, College of Medicine and Health Sciences, University of Gondar, P.O. Box 196, Gondar, Ethiopia; ^2^Department of Medical Laboratory Sciences, School of Biomedical and Laboratory Sciences, College of Medicine and Health Sciences, University of Gondar, Gondar, Ethiopia; ^3^School of Midwifery, College of Medicine and Health Sciences, University of Gondar, Gondar, Ethiopia

## Abstract

**Background:**

Chronic kidney disease is a global health problem with serious adverse effects, including kidney failure, cardiovascular disease, and premature death. Improving awareness and practice on the impact, prevention, and early detection of chronic kidney disease will reduce the significant economic and public health burden.

**Methods:**

A cross-sectional study was conducted to determine knowledge and practice towards prevention and early detection of chronic kidney disease and its associated factors among hypertensive patients in Gondar town in 2019. The study included hypertensive patients visiting health institutions from February to March 2019. Data was collected using a semistructured questionnaire and individuals who fulfilled our inclusion criteria were selected using a systemic random sampling technique. Epi Info software version 7 was used for data entry, and SPSS version 20 was used for descriptive and logistic regression analysis.

**Result:**

Out of a total of 442 participants, 434 completed the questionnaire, with a response rate of 98.1%. Of the total, 298 (68.7%) had good knowledge of chronic kidney disease with a mean knowledge score of 8.78 ± 2.80 and 210 (48.4%) had good practice with mean practice score of 6.58 ± 1.61. Educational status, residence, and duration of hypertension were significantly associated with the knowledge and practice scores of the participants in the multivariate logistic regression analysis.

**Conclusion:**

More than half of the participants had good knowledge about chronic kidney disease and its risk factors. However, the level of preventive practice among participants was low. The educational status, residence, and duration of hypertension were significantly associated variables with knowledge and practice scores in multivariate logistic regression.

## 1. Introduction

Chronic kidney disease (CKD) is a global health problem with an increasing incidence rate, poor outcomes, and high treatment and management cost [1]. CKD is increasing worldwide at an annual growth rate of 8%. There are regional differences in the disease epidemiology; the prevalence of CKD in developing countries is higher than in the developed world [2]. The Global Burden of Disease (GBD), 2015, study estimated that 1.2 million deaths from cardiovascular diseases were directly attributable to reduced glomerular filtration rates (GFR) [3].

CKD can be categorized into five stages, ranging from mild stage-1 disease to severe end-stage renal disease (ESRD), stage 5 [4]. The majority of individuals with CKD are living in low- and middle-income countries. In addition, findings also indicate that women have a higher prevalence of CKD and the prevalence has steadily increased with age in high-income and low-income countries [5].

There are factors that directly cause kidney damage, increase susceptibility to CKD, worsen kidney damage, and result in a faster decline in kidney function. These factors include older age, family history, diabetes mellitus (DM), higher blood pressure, poor glycemic control in diabetes, and smoking [6]. Moreover, the prevalence of CKD increases with increasing age and exceeds 20% in individuals over 60 years of age and 35% in individuals over 70 years of age [6]. Recent data showed that hypertension and DM are the two major causes of kidney disease worldwide, including both developing and developed countries [7].

Hypertension is the second major cause of ESRD besides DM. Nearly, one billion people worldwide have high blood pressure and that number is expected to increase to 1.56 billion people by 2025. The prevalence of hypertension is predicted to increase by 24% and 80% in developed and developing countries, respectively [8]. Hypertensive patients had a far greater prevalence of CKD (26%) compared to those without the disease (8%) [9, 10].

The outcomes of CKD are not only limited to kidney failure (KF) or ESRD but also complications of decreased kidney function or low GFR rate and cardiovascular disease. Recent evidence indicates that early detection and treatment can prevent or delay some of these adverse outcomes [1]. No national studies have been conducted on the prevalence of hypertension in Ethiopia, yet the reported rate of hypertension in some studies varied from 20% to 30% [11].

Increasing awareness among individuals at risk of developing CKD, including those hypertensive patients, about the impact of CKD and its effective management could reduce the significant economic and public health burdens. Knowledge of CKD and risk factors enhances the perception of high risk and increases health-seeking behavior [12]. The implementation of acquired knowledge for prevention and control of CKD, supported by a combination of modern technology and healthcare support for those who are at risk of CKD, will result in good practice [13].

Despite the fact that CKD causes numerous public health problems, there are only a few studies in knowledge and practices towards CKD among high risk individuals, such as hypertensive patients. Thus, there is a need to assess the awareness and preventive practice towards CKD to make an effective intervention plan and to propose an effective policy related for high risk individuals. The aim of the study was to assess the knowledge and preventive practice of hypertensive patients towards CKD and to determine the clinical and sociodemographic factors which may affect the knowledge and practice towards CKD.

## 2. Methods

### 2.1. Study Setting and Design

The study was conducted at Maraki Health Center, Azezo Health Center, and University of Gondar Referral Hospital Chronic Disease Clinic in Gondar town. The institutions are located in the Amhara regional state, which is about 741 kilometers northwest of Addis Ababa, capital city of Ethiopia. Cross-sectional study was conducted to determine the level of knowledge and practice towards CKD and its associated factors among hypertensive patients from February 1 to March 30, 2019.

### 2.2. Population Characteristics

All hypertensive patients followed up at University of Gondar Hospital and Maraki and Azezo Health Center at the time of data collection, who were willing to participate in the study, voluntary provide their sociodemographic information, and were willing to answer questions about knowledge and practice related to CKD were included. Hypertensive patients with present CKD, patients with mental health problems, hearing impairments, or any other critical health problems, and those who are unable to provide the appropriate information were excluded.

### 2.3. Sample Size and Sampling Technique

The sample size was determined by using a single population proportion formula. The proportion was taken at 50%, with 95% of confidence intervals (CI) and 5% of margin error; *n* = *z*^2^p (1 − *p*)/w2, where *n* = sample size, *p* = proportion, *w* = margin error, *z* = 1.96 confidence level, and *n* = 1.96^2^(0.5(1–0.5))/(0.05) (0.05) = 384. By considering the 15% nonresponse rate, the sample size was 442, and a simple random sampling technique was used to select study participants.

Three health facilities, Azezo Health Center, Maraki Health Center, and University of Gondar Specialized Hospital Chronic Clinic, were selected from a total of 11 health institutions identified in Gondar Town using random sampling techniques. Probability proportional to sample size was used to select participants in the health institutions, and every 3^rd^ participant of the study was involved in the study using systematic random sampling techniques from the selected institutions.

### 2.4. Study Instrument

The study instrument was an adaptation of the measures developed in a study done in Palestine and the relevant CKD related scientific literature was reviewed, and, finally, 25 questions were generated to assess knowledge and practice towards CKD [14].

The reliability of the questionnaire was assessed by conducting a pilot study. The prepared questions were pretested to ensure they are eligible and to avoid any confusion regarding the understanding of the questionnaire and the questionnaire was modified based on feedback from the pilot study. A previously developed questionnaire was modified to suit the study setting, the questionnaire was reevaluated after the first pilot study on 11 hypertensive patients, and a further pilot study was performed in another 11 hypertensive patients.

Face validity was performed in hypertensive patients to evaluate the comprehension of the layperson towards understanding of the questionnaire and to assess how important it was to target study participants. Participants were asked to analyze each item of the questionnaire following the open-ended discussion and their diverse responses and interpretation of the different issues; how it was presented and the lack of ambiguity were evaluated. A revised version of the questionnaire was developed on the basis of the face-validation results, which helped to differentiate knowledge and practice items to CKD that were relevant to the study participants. The final draft questionnaire included 3 parts and 34 items (9 items on sociodemographic characteristics, 14 items on knowledge, and 11 items on practice).

Participants who responded correctly to 50% or more of knowledge and practice questions were considered as having adequate knowledge about CKD, whereas those who scored <50% were considered as having poor knowledge and practice towards CKD. Each response was scored as “1” for correct response/practices and “0” for incorrect responses/practices. The knowledge and practice scores of individuals were calculated and summed up to give the total knowledge and practice score.

Data were collected by trained and experienced clinical nurse data collectors through face-to-face interviews using the constructed questionnaires. The questionnaire was first prepared in English and then translated into the local Amharic language with the assistance of a language expert. The questionnaire consisted of three portions: the first portion deals with sociodemographic characteristics; the remaining two portions contain questions on the assessment of participants' knowledge and preventive practice. Data were collected from the selected hypertensive patients by a trained data collector through systematic random sampling and every 3^rd^ participant who fulfilled the inclusion criteria was asked to participate after the aim of the study was briefly explained. The collected data was checked daily for consistency, accuracy, and preventing any bias.

### 2.5. Statistical Analysis

The data were entered by Epi Info software and analyzed by using Statistical Package for Social Science (SPSS) version 20 software. Categorical variables were summarized in frequency, percentage, and chi-square. The data were interpreted by using percentage and chi-square; the result was presented by using tables and graphs. Frequencies and percentages were calculated for all variables related to the study. Binary logistic regression model was used to identify factors associated with knowledge and preventive practice towards CKD. Crude odds ratio (COR) and adjusted odds ratio (AOR) were used to measure the strength of association between dependent and independent variables. In addition, Pearson's correlation between knowledge and practice score was assessed. A *P* value of less than 0.05 was used to classify statistically significant associated variables with the dependent variables.

## 3. Results

### 3.1. Sociodemographic Characteristics of the Study Participants

A total of 442 hypertensive patients were asked to participate in the study and 434 completed the questionnaire and included in the study with a response rate of 98.1%. Out of a total of 434 participants, 220 (50.7%) were male. Majority of the participants, 201 (46.3%), were in the age group >55 years, 117 (27%) were unable to read and write, 156 (35.6%) had DM comorbidity, 290 (66.8%) were married, 359 (82.7%) were living in urban areas, and 287 (66.1%) had < 5-year follow-up (Table 1).

### 3.2. Knowledge of Study Participants

Out of a total of 434 participants, 298 (68.7%) had good knowledge of CKD with the knowledge mean score of 8.78 ± 2.80. Majority of the participants, 267 (61.5%), responded that they heard of CKD at least once before, 279 (64.3%) identified the risk factor of CKD, and 415 (95.6%) knew CKD can be diagnosed and treated. In addition, 372 (85.7%) were aware of the importance of regular checkups for CKD prevention and early detection, but only 155 (35.7%) were aware of the effect of drug overdose and/or traditional medicine on CKD (Table 2).

### 3.3. Preventive Practices of Study Participants

Of the total study participants, 210 (48.4%) had good practice towards CKD with a mean practice score of 6.58 ± 1.61. The majority of the study participants, 379 (87.3%), did not eat well-balanced meals, 402 (92.6%) did not exercise regularly, and 360 (82.9%) did not visit health institutions for diagnosis of other disease comorbidities. However, 418 (96.3%) participants were nonsmokers, 413 (95.2%) correctly followed the medications, and 408 (94.0%) were correctly following food restrictions from physicians (Table 3).

### 3.4. Factors Associated with Knowledge of Study Participants

In multivariate logistic regression, educational status, residence, and duration of hypertension were significantly associated with knowledge towards CKD. Accordingly, urban resident study participants were 2.21 times (AOR = 2.21; 95% CI: 1.09, 4.5) knowledgeable compared to rural resident participants. In addition, the study participants who attended primary and secondary schools were 18.34 times (AOR = 18.34; 95% CI: 2.1, 16.7) and 8.6 times (AOR = 8.6; 95% CI: 2.11, 34.99) knowledgeable than those who could not read and write, respectively. In addition, the study participants with a duration of 5 to 10 years of hypertension were 2.14 times knowledgeable (AOR = 2.14; 95% CI: 1.22, 3.75) compared to participants with fewer than 5 years of hypertension duration (Table 4).

### 3.5. Factors Associated with Preventive Practice of the Study Participants

In multivariate logistic regression, educational status, residence, and duration of hypertension were significantly associated with level of practice towards CKD. Therefore, urban residents and college and above educational status were 2.27 times (AOR = 2.27; 95% CI: 1.06, 4.83) and 6.26 times (AOR = 6.26; 95% CI: 3.35, 20.37) higher preventive practices compared to rural residents and unable to read and write participants, respectively. In addition, the study participants with a duration of fewer than five years of hypertension have an 81% higher preventive practice than participants with a duration of more than 15 years of hypertension (Table 5).

## 4. Discussion

Of the 434 participants, 298 (68.7%) had good knowledge of CKD. This finding was higher than the study done in Nigeria (27.1%) [12], Malaysia (26.3%) [15] and (30.1%) [16], and Palestine (61.8%). The difference may be because the study participants were institution-based and had a better access to health education than the general community. However, this finding was lower compared to the study done in Jordan, where 80.24% were knowledgeable towards CKD [17]. This might be due to the difference in sample size and source population of the study.

More than half of the participants 267 (61.5%) heard of CKD at least once before. This finding was slightly lower than the study done in Malaysia (69.5%) [15]. The difference may be due to difference in access to health education of the participant. In the current study, 275 (63.4%) correctly answered the signs and symptoms of CKD; this finding was higher than the Jordan study in which about half of the participants 370 (50%) had incorrect information about CKD signs and symptoms [17]. The possible reason for the variation might be attributed to the differences in sociodemographic and access to CKD learning opportunities.

In the current study, 244 (56.2%) of the participants were aware of the effect of uncontrolled hypertension on CKD, which was higher than the study in Tanzania, where only 17% were aware that high blood pressure could cause CKD [18]. This may be due to differences in access to health education. In addition, the study found that 244 (56.2%) were aware of uncontrolled hypertension on CKD, which was higher than the study conducted in Iran that only 14.4% of the respondents were aware of the uncontrolled hypertension as very likely to result in CKD [19]. In addition, 279 (64.3%) of participants responded correctly to the risk factors of CKD and this was higher in the Jordan study, with more than 50% of the participants identifying the risk factors of CKD [17]. This may be due to differences in the study population since because our study participants were on medical follow-up and had a health education program, Iran and Jordan's studies were community based.

Of the study participants, 48.4% had good practice towards CKD. This finding was lower compared to the studies conducted in a tertiary teaching hospital, Malaysia, which found good practice scores in more than 88.3% of participants [16]. This may be due to difference in educational status, economic status, and health education practice about CKD. Out of a total of participants, 12.7% take well-balanced diet, which was lower than the Jordan study, 60.6% [17]. This may be due to differences in the economic status of study participants. However, 95.2% of the participants adhered to medication, which was higher than the Jordan study of 62.7% [17]. This may be due to the fact that the current study's participants have been included from the health institution and that there are regular follow-up and health education opportunities for the patients.

In the multivariate logistic analysis, knowledge score was significantly associated with the educational status of our study participants; this was similar to the study in Tanzania [18] and Jordan [17]. This was because participants who had higher educational status had more knowledge about hypertension, its risk, and ways to prevent its adverse effect. In addition, our study showed that there was no significant association between age and knowledge score, which varied from the study done in Brazil [20]. This may be due to disparity in sociodemographic characteristics of the study participants.

In multivariate logistic regression, educational status and duration of hypertension were statistically significantly associated with a higher practice score in multivariate logistic regression. This finding was consistent with the study done in Palestine [14]. This may be due to the fact that participants with higher education levels have access to information from different journals and information sources. However, we found that there was no significant association between age and practice. This finding was different from the study conducted in Jordan, which showed that being of older age is associated with a better practice score [17]. This may be due to differences in sample size and sociodemographic characteristics of the study participants.

### 4.1. Limitation

The response of the participants may be affected by the interviewer's bias and the findings of this study cannot be generalized to the other city populations of the country due to the difference in sociodemographic characteristics.

## 5. Conclusion

More than half of the participants had good knowledge of CKD. However, fewer than half of the participants had a good preventive practice score towards the CKD. In multivariate logistic regression, educational status, residence, and duration of hypertension were significantly associated with knowledge and practice score of participants towards CKD. Therefore, additional effort should be made by responsible organizations to increase the community's awareness and preventive practice towards CKD.

## Figures and Tables

**Table 1:** Sociodemographic characteristics of the study participants at Gondar town in 2019.

Variable	Categories	Frequency (*n*)	Percentage (%)
Age (years)	<35	40	9.2
	36–45	101	23.3
	46–55	92	21.2
	>55	201	46.3

Sex	Male	220	50.7
	Female	214	49.3

Marital status	Single	63	14.5
	Divorced	54	12.4
	Married	290	66.8
	Widowed	27	6.2

Educational status	Unable to read and write	117	27.0
	Able to read and write without formal education	169	38.9
	Primary school	23	5.3
	Secondary school	41	9.4
	College/university and above	84	19.4

Religion	Orthodox	353	81.3
	Protestant	20	4.6
	Muslim	60	13.6
	Others	1	0.2

Residence	Urban	359	82.7
	Rural	75	17.3

Occupation	Private business	74	17.1
	Government employee	89	20.5
	Private employee	88	20.3
	Daily laborer	53	12.2
	Student	24	5.5
	Unemployed	86	19.8
	Others	20	4.6

Duration of hypertension (years)	Less than 5	287	66.1
	5 to 15	122	28.1
	Above 15	25	5.7

Disease comorbidities	Diabetes mellitus	156	35.9
	Stroke	48	11.1
	Asthma	25	5.8
	HIV/AIDS	3	0.7
	Hypercholesterolemia	4	0.9
	Malaria	23	5.3
	Tuberculosis	12	2.8
	Other	17	3.9
	None	153	35.2

**Table 2:** Knowledge assessment towards CKD among hypertensive patients at Gondar town in 2019.

Knowledge assessment	Correct response *n* (%)	Incorrect response *n* (%)
Ever heard of CKD	267 (61.5)	167 (38.5)
Risk factors of CKD	279 (64.3)	155 (35.7)
Effect of prolonged use of medications on CKD	191 (44.0)	243 (56.0)
Effect of uncontrolled hypertension on CKD	244 (56.2)	190 (43.8)
Effect of poor glycemic control on CKD	173 (39.9)	261 (60.1)
Effect of early detection of CKD	168 (38.7)	266 (61.3)
Effect of unprescribed traditional drugs on CKD	155 (35.7)	279 (64.3)
CKD can be diagnosed and treated	415 (95.6)	19 (4.4)
Mentioned at least one sign and symptom of CKD	275 (63.4)	159 (36.6)
Effect of CKD on other diseases	326 (75.1)	108 (24.9)
Effect of regular exercise on CKD	246 (56.7)	188 (43.3)
Effect of smoking on CKD	323 (74.4)	111 (25.6)
Effect of regular blood pressure follow-up and control on CKD	372 (85.7)	62 (14.3)
Effect of excessive alcohol drinking on CKD	378 (87.1)	56 (12.9)

CKD : chronic kidney disease.

**Table 3:** Practice assessment towards CKD among hypertensive patients at Gondar town in 2019.

Practice assessment	Good *n* (%)	Poor *n* (%)
Balanced diet	55 (12.7)	379 (87.3)
Regular exercise	32 (7.4)	402 (92.6)
Smoke cigarette	16 (3.7)	418 (96.3)
Drink alcohol	319 (73.5)	115 (26.5)
Normal body mass index	217 (50.0)	217 (50.0)
Regular blood pressure checkup	389 (89.6)	45 (10.4)
Take traditional medicine without physician recommendation	214 (49.3)	220 (50.7)
Follow hypertension medications regimen/treatment adherence	413 (95.2)	21 (4.8)
Follow food restrictions by the physicians	408 (94.0)	26 (6.0)
Personal hygiene	315 (72.6)	119 (27.4)
Regular visit to health institutions for diagnosis of CKD	74 (17.1)	360 (82.9)

CKD : chronic kidney disease.

**Table 4:** Bivariate and multivariate analysis of factors associated with knowledge towards CKD.

Variables	Category	Knowledge		COR 95% CI	AOR 95% CI	*P*value
		Good *n* (%)	Poor *n* (%)			
Sex	Male	166 (38.2%)	54 (12.4%)	1.00	1.00	
	Female	132 (30.4)	82 (18.8%)	0.524 (0.35,0.79)	0.61 (0.36,1.01)	0.055

Educational status	Unable to read and write	60 (13.8%)	57 (13.1%)	1.00	1.00	
	Able to read and write without formal education	113 (26.0)	56 (12.9%)	1.917 (1.18,3.11)	1.29 (0.68,2.45)	0.223
	Primary school	22 (5.0%)	1.0 (0.23%)	20.9(2.73,160.20)	18.34(2.1,16.70)	0.008^*∗*^
	Secondary school	38 (8.7%)	3.0 (0.69%)	12.03 (3.52,41.17)	8.6 (2.11,34.99)	0.003^*∗*^
	University/college and above	65 (14.9%)	19 (4.3%)	3.25 (1.74,6.08)	1.86 (0.77,4.50)	0.168

Residence	Rural	40 (9.2%)	35 (8.0%)	1.00	1.00	
	Urban	258 (59.4%)	101 (23%)	2.235 (1.34,3.72)	2.21 (1.09,4.50)	0.028^*∗*^

Hypertension duration (years)	<5	186 (42.8%)	101 (23%)	1.00	1.00	
	5–15	92 (21.1%)	30 (6.9%)	1.66 (1.03,2.69)	2.14 (1.22,3.75)	0.008^*∗*^
	>15	20 (4.6%)	5.0 (1.1%)	2.17 (0.79,5.96)	3.02 (0.97,9.40)	0.056

Age group (years)	<35	24 (5.5%)	16 (3.6%)	1.00	1.00	
	35–45	71 (16.3%)	31 (7.1%)	1.53 (0.71,3.27)	1.21(0.5,2.90)	0.666
	45–55	64 (14.7%)	28 (6.4%)	1.524 (0.7,3.30)	1.42 (0.58,3.48)	0.442
	>55	139 (32%)	61 (14%)	1.53 (0.75,3.06)	1.55 (0.68,3.50)	0.291

AOR = adjusted odds ratio, COR = crude odds ratio, CI = confidence interval, ^*∗*^ = *P* < 0.05 significant association.

**Table 5:** Bivariate and multivariate analysis of factors associated with preventive practice towards CKD.

Variables	Category	Practice		COR 95% CI	AOR 95% CI	*P* value
		Good *n* (%)	Poor *n* (%)			
Sex	Male	91 (20.9%)	129 (29.7%)	1.00	1.00	
	Female	119 (27.4%)	95 (21.8%)	1.78 (1.21,2.60)	1.65 (0.99,2.71)	0.05

Age	<35 years	19 (4.3%)	21 (4.8%)	1.00	1.00	
	35–45 years	48 (11.0%)	54 (12.4%)	0.98 (0.47,2.04)	0.79 (0.325,1.93)	0.609
	45–55 years	41 (9.4%)	51 (11.7%)	0.89 (0.42,1.87)	0.68 (0.27,1.69)	0.408
	>55 years	102 (23.5%)	98 (22.5%)	1.15 (0.58,2.27)	0.89 (0.39,2.05)	0.784

Educational status	Illiterates	49 (11.2%)	68 (15.6%)	1.00	1.00	
	Able read and write	72 (16.5%)	97 (22.3%)	1.03 (0.64,1.66)	1.51 (0.77,2.96)	0.230
	Primary school	8 (1.8%)	15 (3.4%)	0.74 (0.29,1.88)	1.46 (0.46,4.61)	0.523
	Secondary school	18 (4.1%)	23 (5.2%)	1.086(0.53,2.23)	2.47 (0.91,6.76)	0.077
	University/college and above	63 (14.5%)	21 (4.8%)	4.16 (2.25,7.70)	8.26(3.35,20.37)	<0.001∗

Marital status	Married	145 (33.4)	145 (33.4%)	1.00	1.00	
	Divorced	32 (7.3%)	22 (5.0%)	1.45 (0.81,2.62)	1.47 (0.675,3.20)	0.332
	Widowed	9 (2.0%)	18 (4.1%)	0.50 (0.22,1.15)	0.30 (0.104,1.01)	0.058
	Single	24 (5.5%)	39 (8.9%)	0.61 (0.35,1.07)	0.55 (0.26,1.16)	0.118

Residence	Rural	18 (4.1%)	57 (13.1%)	1.00	1.00	
	Urban	192 (44.2%)	167 (38.4%)	3.64 (2.06,6.43)	2.27 (1.06,4.83)	0.034^*∗*^

Hypertension duration (years)	<5 years	132 (30.4%)	155 (35.5%)	1.00	1.00	
	5–15 years	70 (16.1%)	52 (11.9%)	1.58 (1.03,2.42)	1.41 (0.84,2.38)	0.197
	>15 years	8 (1.8%)	17 (3.9%)	0.55(0.21,1.32)	0.19 (0.065,0.58)	0.003∗

AOR = adjusted odds ratio, COR = crude odds ratio, CI = confidence interval ^*∗*^ = *P* < 0.05 significant association.

## Data Availability

Most of the data generated or analyzed during this study are included in this published article, and additional files will be available from the corresponding author upon request.

## Abbreviations

AOR: Adjusted odds ratio
BP: Blood pressure
CI: Confidence interval
CKD: Chronic kidney disease
COR: Crude odds ratio
DM: Diabetes mellitus
ESRD: End-stage renal disease
GBD: Global Burden of Disease
GFR: Glomerular filtration rate
SPSS: Statistical Package for Social Science.

## Supplementary Materials

Supplementary Materials English version questionnaires. Part 1: sociodemographic information of participants. Part 2: knowledge assessment questionnaires. Part 3: questionnaires to assess practice.
