# Supplementary material for: Knowledge and Practices towards Prevention and Early Detection of Chronic Kidney Disease and Associated Factors among Hypertensive Patients in Gondar Town, North West Ethiopia
Source: Int J Hypertens. 2020 Aug 6;2020:2860143. doi: 10.1155/2020/2860143 (PMC7428870; doi:10.1155/2020/2860143)
Supplement: Supplementary Materials — English version questionnaires. Part 1: sociodemographic information of participants. Part 2: knowledge assessment questionnaires. Part 3: questionnaires to assess practice. [file 2860143.f1.docx]

**English version Questionnaires**

**Part 1: Socio-demographic information of participants**

| N^o^ | Questions | Answers |
| --- | --- | --- |
| **101** | Age in Years | ----------- |
| **102** | Sex | 1. Male 2. Female |
| **103** | Educational status | 1. Unable to read and write 2. read and write 3. primary school 4. secondary school 5. college or University |
| **104** | Marital status | 1. Single 2. Married 3. widowed 4. Divorced 5. Other specify……………… |
| **105** | Residence | 1. Urban 2. Rural |
| **106** | Occupation | 1. Private business 2. Government employee 3. Private employee 4. Daily laborer 5. Student 6. Unemployed 7. Others |
| **107** | Religion | 1. Orthodox 2. Protestant 3. Muslim 4. Catholic 5. Others specify……………… |
| **108** | Chronic diseases other than hypertension | 1. Diabetes mellitus 2. Stroke 3. Asthma 4. HIV/AIDS 5. Hypercholesterolemia 6. Malaria 7. Tuberculosis 8. Other 9. None |
| **109** | Hypertension duration (In years) | ------------------ |

**Part: 2** Knowledge assessment questionnaires

| No | Questions | Answers |
| --- | --- | --- |
| **201** | Have you ever heard of CKD? | 1. Yes 2. No |
| **202** | Do you know the risk factors of CKD? | 1. Correct Answer 2. Incorrect Answer |
| **203** | Do you know the effect of prolonged use of medications on CKD? | 1. Correct Answer 2. Incorrect Answer |
| **204** | Do you know the effect of uncontrolled hypertension on CKD? | 1. Correct Answer 2. Incorrect Answer |
| **205** | Do you know the effect of poor glycemic control on CKD? | 1. Correct Answer 2. Incorrect Answer |
| **206** | Do you know the effect of early detection of CKD? | 1. Correct Answer 2. Incorrect Answer |
| **207** | Do you know the effect of unprescribed traditional drugs on CKD? | 1. Correct Answer 2. Incorrect Answer |
| **208** | Do you know CKD can be diagnosed and treated? | 1. Correct Answer 2. Incorrect Answer |
| **209** | Do you know the sign and symptom of CKD? | 1. Correct Answer 2. Incorrect Answer |
| **210** | Do you know the effect of CKD on other diseases or body organs? | 1. Correct Answer 2. Incorrect Answer |
| **211** | Do you know the effect of regular exercise on hypertension? | 1. Correct Answer 2. Incorrect Answer |
| **212** | Do you know the effect of smoking on chronic kidney disease? | 1. Correct Answer 2. Incorrect Answer |
| **213** | Do you know the effect of excessive alcohol drinking on CKD? | 1. Correct Answer 2. Incorrect Answer |
| **214** | Do you know the the effect of regular blood pressure control on CKD? | 1. Correct Answer 2. Incorrect Answer |

**Part: 3** Questionnaires to Assess Practice

| **301** | Do you eat well-balanced meals recommended by the physicians? | 1. Yes 2. No |
| --- | --- | --- |
| **302** | Do you perform regular exercise? | 1. Yes 2. No |
| **303** | If your answer to question 2 is yes  How many times you perform regular exercise per week? | 1. Every day 2. 2-3 times per week 3. Randomly 4. Any other |
| **304** | Do you have regular blood pressure follow up? | 1. Yes 2. No |
| **305** | Do you maintain healthy body weight (Body mass index)? | 1. Yes 2. No |
| **306** | Do you have a regular follow up/diagnosis to early detection of CKD? | 1. Yes 2. No |
| **307** | Do you smoke cigarette? | 1. Yes 2. No |
| **308** | Do you follow your medication regimen or treatment? | 1. Yes 2. No |
| **309** | Do you correctly follow your food restrictions, such as low salt diet and diabetic diet, etc.? | 1. Yes 2. No |
| **310** | Do you keep your personal hygiene? | 1. Yes 2. No |
| **311** | Do you take medical and traditional treatments without physicians’ recommendation? | 1. Yes 2. No |
